# 2-[(*E*)-(2,3-Dimethyl­phen­yl)imino­meth­yl]phenol

**DOI:** 10.1107/S1600536810033398

**Published:** 2010-08-28

**Authors:** M. Nawaz Tahir, Muhammad Ilyas Tariq, Shahbaz Ahmad, Muhammad Sarfraz, Riaz H. Tariq

**Affiliations:** a Department of Physics, University of Sargodha, Sargodha, Pakistan; b Department of Chemistry, University of Sargodha, Sargodha, Pakistan; c Institute of Chemical and Pharmaceutical Sciences, The University of Faisalabad, Faisalabad, Pakistan

## Abstract

In the title compound, C_15_H_15_NO, the almost planar 2,3-dimethyl­aniline unit and the salicyl­aldehyde group (r.m.s. deviations of 0.0156 and 0.0109 Å, respectively) are oriented at a dihedral angle of 43.69 (9)° with respect to each other. An *S*(6) ring motif is formed due to intra­molecular O—H⋯N hydrogen bonding. In the crystal, C—H⋯π inter­actions occur between the 2,3-dimethyl­aniline unit and the salicyl­aldehyde group, where the CH is from the *o*-methyl group.

## Related literature

For background to Schiff bases synthesized from 2,3-dimethyl­aniline and for related structures, see: Tahir *et al.* (2010*a* ▶,*b* ▶); Tariq *et al.* (2010 ▶). For graph-set notation, see: Bernstein *et al.* (1995 ▶).
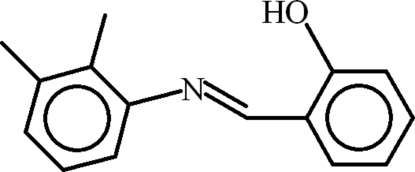


## Experimental

### 

#### Crystal data

C_15_H_15_NO
*M* *_r_* = 225.28
Orthorhombic, 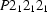

*a* = 7.5641 (7) Å
*b* = 12.5889 (13) Å
*c* = 13.0643 (14) Å
*V* = 1244.0 (2) Å^3^
*Z* = 4
Mo *K*α radiation
μ = 0.08 mm^−1^
*T* = 296 K
0.32 × 0.12 × 0.10 mm

#### Data collection

Bruker Kappa APEXII CCD diffractometer
Absorption correction: multi-scan (*SADABS*; Bruker, 2005 ▶) *T*_min_ = 0.980, *T*_max_ = 0.985
9729 measured reflections
1316 independent reflections
687 reflections with *I* > 2σ(*I*)
*R*_int_ = 0.085

#### Refinement

*R*[*F* ^2^ > 2σ(*F* ^2^)] = 0.078
*wR*(*F* ^2^) = 0.157
*S* = 1.11
1316 reflections
157 parameters
H-atom parameters constrained
Δρ_max_ = 0.11 e Å^−3^
Δρ_min_ = −0.13 e Å^−3^

### 

Data collection: *APEX2* (Bruker, 2009 ▶); cell refinement: *SAINT* (Bruker, 2009 ▶); data reduction: *SAINT*; program(s) used to solve structure: *SHELXS97* (Sheldrick, 2008 ▶); program(s) used to refine structure: *SHELXL97* (Sheldrick, 2008 ▶); molecular graphics: *ORTEP-3 for Windows* (Farrugia, 1997 ▶) and *PLATON* (Spek, 2009 ▶); software used to prepare material for publication: *WinGX* (Farrugia, 1999 ▶) and *PLATON*.

## Supplementary Material

Crystal structure: contains datablocks global, I. DOI: 10.1107/S1600536810033398/bq2232sup1.cif

Structure factors: contains datablocks I. DOI: 10.1107/S1600536810033398/bq2232Isup2.hkl

Additional supplementary materials:  crystallographic information; 3D view; checkCIF report

## Figures and Tables

**Table 1:** Hydrogen-bond geometry (Å, °) *Cg*1 is the centroid of the C1–C6 ring.

*D*—H⋯*A*	*D*—H	H⋯*A*	*D*⋯*A*	*D*—H⋯*A*
O1—H1⋯N1	0.82	1.85	2.582 (6)	148
C7—H7*A*⋯*Cg*1^i^	0.96	2.92	3.782 (6)	150

Symmetry code: (i) 
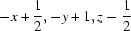
.

**Fig. 1.**
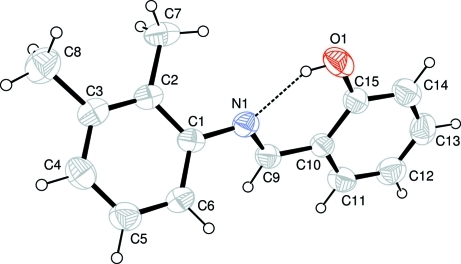
View of the title compound with the atom numbering scheme. The thermal ellipsoids are drawn at the 30% probability level. The dotted line represents the intramolecular H-bonding.

## supplementary crystallographic information

### Comment

We have reported crystal structures of Schiff bases synthesized from 2,3-dimethylaniline (Tahir *et al.*, 2010*a*, 2010*b*), (Tariq *et al.*, 2010) and in continuation of this work, we report herein the structure and synthesis of the title compound (I, Fig. 1). The title compound has been synthesized for the preparation of different organometallic compounds.

In (I), the 2,3-dimethylaniline moiety A (C1–C8/N1) and the group B (C9—C15/O1) of salicylaldehyde are planar with r.m.s. deviations of 0.0156 and 0.0109 Å, respectively. The dihedral angle between A/B is 43.69 (9)°. The title molecule essentially consists of monomers. In the title molecule an S(6) ring motif (Bernstein *et al.*, 1995) is formed due to intramolecular H-bonding of O—H···N type (Table 1, Fig. 1). There exist C—H···π interaction (Table 1) which plays important role in stabilizing the molecules.

### Experimental

Equimolar quantities of 2,3-dimethylaniline and salicylaldehyde were refluxed in methanol for 45 min. The resulting solution was kept at room temperature which afforded colorless needles of (I) after 72 h.

### Refinement

The H-atoms were positioned geometrically (O–H = 0.82, C–H = 0.93–0.96 Å) and refined as riding with *U*_iso_(H) = *xU*_eq_(C), where *x* = 1.5 for methyl and *x* = 1.2 for all other H-atoms. In the absence of significant anomalous scattering factor, Friedal pairs were merged.

### Crystal data

**Table:**

C_15_H_15_NO	*F*(000) = 480
*M**_r_* = 225.28	*D*_x_ = 1.203 Mg m^−^^3^
Orthorhombic, *P*2_1_2_1_2_1_	Mo *K*α radiation, λ = 0.71073 Å
Hall symbol: P 2ac 2ab	Cell parameters from 687 reflections
*a* = 7.5641 (7) Å	θ = 2.3–25.2°
*b* = 12.5889 (13) Å	µ = 0.08 mm^−^^1^
*c* = 13.0643 (14) Å	*T* = 296 K
*V* = 1244.0 (2) Å^3^	Needle, colorless
*Z* = 4	0.32 × 0.12 × 0.10 mm

### Data collection

**Table:**

Bruker Kappa APEXII CCD diffractometer	1316 independent reflections
Radiation source: fine-focus sealed tube	687 reflections with *I* > 2σ(*I*)
graphite	*R*_int_ = 0.085
Detector resolution: 8.1 pixels mm^-1^	θ_max_ = 25.2°, θ_min_ = 2.3°
ω scans	*h* = −9→8
Absorption correction: multi-scan (*SADABS*; Bruker, 2005)	*k* = −15→15
*T*_min_ = 0.980, *T*_max_ = 0.985	*l* = −15→15
9729 measured reflections	

### Refinement

**Table:**

Refinement on *F*^2^	Primary atom site location: structure-invariant direct methods
Least-squares matrix: full	Secondary atom site location: difference Fourier map
*R*[*F*^2^ > 2σ(*F*^2^)] = 0.078	Hydrogen site location: inferred from neighbouring sites
*wR*(*F*^2^) = 0.157	H-atom parameters constrained
*S* = 1.11	*w* = 1/[σ^2^(*F*_o_^2^) + (0.0624*P*)^2^] where *P* = (*F*_o_^2^ + 2*F*_c_^2^)/3
1316 reflections	(Δ/σ)_max_ < 0.001
157 parameters	Δρ_max_ = 0.11 e Å^−^^3^
0 restraints	Δρ_min_ = −0.13 e Å^−^^3^

### Special details

**Table:**

Geometry. Bond distances, angles *etc*. have been calculated using the rounded fractional coordinates. All su's are estimated from the variances of the (full) variance-covariance matrix. The cell e.s.d.'s are taken into account in the estimation of distances, angles and torsion angles
Refinement. Refinement of *F*^2^ against ALL reflections. The weighted *R*-factor *wR* and goodness of fit *S* are based on *F*^2^, conventional *R*-factors *R* are based on *F*, with *F* set to zero for negative *F*^2^. The threshold expression of *F*^2^ > σ(*F*^2^) is used only for calculating *R*-factors(gt) *etc*. and is not relevant to the choice of reflections for refinement. *R*-factors based on *F*^2^ are statistically about twice as large as those based on *F*, and *R*- factors based on ALL data will be even larger.

### Fractional atomic coordinates and isotropic or equivalent isotropic displacement parameters (Å^2^)

**Table:**

	*x*	*y*	*z*	*U*_iso_*/*U*_eq_	
O1	−0.1515 (5)	0.5069 (3)	0.2310 (3)	0.0897 (19)	
N1	−0.0156 (5)	0.3687 (4)	0.1080 (3)	0.0627 (17)	
C1	0.0128 (6)	0.3279 (4)	0.0080 (4)	0.056 (2)	
C2	0.0758 (6)	0.3945 (5)	−0.0690 (5)	0.059 (2)	
C3	0.0952 (6)	0.3556 (5)	−0.1668 (4)	0.065 (2)	
C4	0.0551 (7)	0.2503 (6)	−0.1881 (5)	0.077 (3)	
C5	−0.0066 (7)	0.1849 (5)	−0.1113 (5)	0.083 (3)	
C6	−0.0298 (7)	0.2231 (5)	−0.0142 (5)	0.072 (2)	
C7	0.1219 (8)	0.5080 (4)	−0.0424 (5)	0.088 (3)	
C8	0.1642 (9)	0.4236 (5)	−0.2532 (4)	0.106 (3)	
C9	0.0214 (6)	0.3121 (4)	0.1866 (4)	0.064 (2)	
C10	−0.0210 (7)	0.3453 (4)	0.2888 (4)	0.061 (2)	
C11	0.0197 (8)	0.2804 (5)	0.3715 (4)	0.081 (2)	
C12	−0.0244 (10)	0.3084 (6)	0.4696 (5)	0.095 (3)	
C13	−0.1103 (10)	0.4022 (7)	0.4868 (6)	0.097 (3)	
C14	−0.1526 (7)	0.4687 (5)	0.4063 (6)	0.088 (3)	
C15	−0.1088 (7)	0.4403 (5)	0.3083 (5)	0.068 (3)	
H1	−0.11767	0.48168	0.17655	0.1075*	
H4	0.06981	0.22390	−0.25406	0.0926*	
H5	−0.03254	0.11425	−0.12564	0.0997*	
H6	−0.07406	0.17895	0.03678	0.0859*	
H7A	0.24802	0.51622	−0.04157	0.1311*	
H7B	0.07492	0.52507	0.02387	0.1311*	
H7C	0.07187	0.55492	−0.09262	0.1311*	
H8A	0.08868	0.48428	−0.26179	0.1586*	
H8B	0.16581	0.38290	−0.31528	0.1586*	
H8C	0.28187	0.44699	−0.23737	0.1586*	
H9	0.07767	0.24716	0.17715	0.0767*	
H11	0.07817	0.21654	0.36011	0.0969*	
H12	0.00398	0.26397	0.52398	0.1147*	
H13	−0.14067	0.42136	0.55328	0.1166*	
H14	−0.21052	0.53261	0.41862	0.1054*	

### Atomic displacement parameters (Å^2^)

**Table:**

	*U*^11^	*U*^22^	*U*^33^	*U*^12^	*U*^13^	*U*^23^
O1	0.084 (3)	0.075 (3)	0.110 (4)	0.023 (3)	0.003 (3)	−0.009 (3)
N1	0.049 (3)	0.061 (3)	0.078 (3)	−0.008 (2)	0.003 (3)	−0.001 (3)
C1	0.038 (3)	0.050 (4)	0.081 (4)	−0.001 (3)	−0.005 (3)	−0.001 (3)
C2	0.045 (3)	0.057 (4)	0.075 (4)	−0.002 (3)	−0.009 (3)	0.010 (4)
C3	0.052 (3)	0.071 (5)	0.073 (4)	0.001 (3)	−0.015 (3)	0.018 (4)
C4	0.071 (4)	0.083 (5)	0.077 (4)	0.005 (3)	−0.009 (3)	−0.015 (4)
C5	0.078 (4)	0.071 (5)	0.101 (5)	−0.011 (4)	0.004 (4)	−0.010 (4)
C6	0.071 (4)	0.063 (4)	0.081 (4)	−0.012 (3)	0.009 (3)	0.005 (3)
C7	0.084 (4)	0.057 (4)	0.122 (5)	−0.011 (4)	−0.007 (4)	0.015 (4)
C8	0.118 (6)	0.113 (6)	0.086 (5)	−0.006 (5)	−0.005 (4)	0.019 (4)
C9	0.049 (3)	0.056 (4)	0.087 (4)	0.001 (3)	0.005 (3)	−0.003 (4)
C10	0.055 (3)	0.057 (4)	0.071 (4)	−0.003 (3)	0.004 (3)	−0.005 (3)
C11	0.090 (4)	0.066 (4)	0.086 (4)	−0.004 (4)	−0.002 (4)	−0.007 (4)
C12	0.114 (6)	0.089 (6)	0.083 (5)	−0.024 (5)	0.003 (4)	−0.003 (4)
C13	0.084 (5)	0.112 (7)	0.096 (5)	−0.030 (5)	0.019 (4)	−0.031 (5)
C14	0.067 (4)	0.078 (5)	0.118 (6)	−0.001 (3)	0.003 (4)	−0.018 (5)
C15	0.054 (3)	0.065 (5)	0.085 (5)	−0.003 (3)	0.005 (3)	−0.006 (4)

### Geometric parameters (Å, °)

**Table:**

O1—C15	1.352 (7)	C13—C14	1.382 (11)
O1—H1	0.8200	C14—C15	1.370 (10)
N1—C1	1.420 (7)	C4—H4	0.9300
N1—C9	1.281 (7)	C5—H5	0.9300
C1—C6	1.389 (8)	C6—H6	0.9300
C1—C2	1.394 (8)	C7—H7A	0.9600
C2—C7	1.511 (8)	C7—H7B	0.9600
C2—C3	1.376 (8)	C7—H7C	0.9600
C3—C4	1.388 (10)	C8—H8A	0.9600
C3—C8	1.510 (8)	C8—H8B	0.9600
C4—C5	1.379 (9)	C8—H8C	0.9600
C5—C6	1.368 (9)	C9—H9	0.9300
C9—C10	1.435 (7)	C11—H11	0.9300
C10—C15	1.392 (8)	C12—H12	0.9300
C10—C11	1.389 (8)	C13—H13	0.9300
C11—C12	1.370 (9)	C14—H14	0.9300
C12—C13	1.366 (11)		
			
O1···N1	2.582 (6)	H1···H7B	2.5300
O1···H8C^i^	2.8900	H4···H8B	2.2700
N1···O1	2.582 (6)	H5···C8^v^	3.0400
N1···H1	1.8500	H5···H8A^v^	2.2400
N1···H7B	2.3600	H6···C9	2.6800
C1···C6^ii^	3.520 (7)	H6···H9	2.3300
C2···C6^ii^	3.503 (8)	H6···C2^iii^	2.8400
C6···C2^iii^	3.503 (8)	H6···C3^iii^	3.0600
C6···C1^iii^	3.520 (7)	H7A···C8	3.0700
C1···H1	3.0900	H7A···C12^vii^	3.0400
C2···H6^ii^	2.8400	H7A···C13^vii^	2.9500
C2···H14^iv^	2.9200	H7B···N1	2.3600
C3···H6^ii^	3.0600	H7B···H1	2.5300
C5···H8A^v^	3.0800	H7C···C8	2.7600
C6···H9	2.6500	H7C···H8A	2.3900
C7···H8A	2.8900	H8A···C7	2.8900
C7···H8C	2.9200	H8A···H7C	2.3900
C8···H7A	3.0700	H8A···C5^vi^	3.0800
C8···H7C	2.7600	H8A···H5^vi^	2.2400
C8···H5^vi^	3.0400	H8B···H4	2.2700
C9···H1	2.3800	H8C···C7	2.9200
C9···H6	2.6800	H8C···O1^vii^	2.8900
C12···H7A^i^	3.0400	H8C···C15^vii^	2.9100
C13···H7A^i^	2.9500	H9···C6	2.6500
C15···H8C^i^	2.9100	H9···H6	2.3300
H1···N1	1.8500	H9···H11	2.4200
H1···C1	3.0900	H11···H9	2.4200
H1···C9	2.3800	H14···C2^viii^	2.9200
			
C15—O1—H1	109.00	C4—C5—H5	120.00
C1—N1—C9	120.2 (5)	C6—C5—H5	120.00
N1—C1—C2	119.9 (5)	C1—C6—H6	120.00
C2—C1—C6	120.0 (5)	C5—C6—H6	120.00
N1—C1—C6	120.0 (5)	C2—C7—H7A	110.00
C1—C2—C3	119.5 (6)	C2—C7—H7B	109.00
C1—C2—C7	118.8 (5)	C2—C7—H7C	109.00
C3—C2—C7	121.7 (6)	H7A—C7—H7B	109.00
C2—C3—C4	120.2 (5)	H7A—C7—H7C	109.00
C2—C3—C8	122.0 (5)	H7B—C7—H7C	109.00
C4—C3—C8	117.9 (5)	C3—C8—H8A	109.00
C3—C4—C5	119.9 (6)	C3—C8—H8B	109.00
C4—C5—C6	120.5 (6)	C3—C8—H8C	109.00
C1—C6—C5	119.9 (6)	H8A—C8—H8B	109.00
N1—C9—C10	122.3 (5)	H8A—C8—H8C	109.00
C9—C10—C15	121.8 (5)	H8B—C8—H8C	110.00
C11—C10—C15	118.0 (5)	N1—C9—H9	119.00
C9—C10—C11	120.2 (5)	C10—C9—H9	119.00
C10—C11—C12	121.5 (6)	C10—C11—H11	119.00
C11—C12—C13	119.5 (7)	C12—C11—H11	119.00
C12—C13—C14	120.6 (7)	C11—C12—H12	120.00
C13—C14—C15	119.8 (6)	C13—C12—H12	120.00
O1—C15—C14	118.6 (5)	C12—C13—H13	120.00
C10—C15—C14	120.7 (6)	C14—C13—H13	120.00
O1—C15—C10	120.7 (5)	C13—C14—H14	120.00
C3—C4—H4	120.00	C15—C14—H14	120.00
C5—C4—H4	120.00		
			
C9—N1—C1—C2	−141.7 (5)	C3—C4—C5—C6	0.4 (8)
C9—N1—C1—C6	41.3 (7)	C4—C5—C6—C1	−1.5 (8)
C1—N1—C9—C10	−173.7 (4)	N1—C9—C10—C11	179.1 (5)
N1—C1—C2—C7	3.3 (7)	N1—C9—C10—C15	1.5 (8)
C6—C1—C2—C3	0.0 (7)	C9—C10—C11—C12	−177.8 (6)
C6—C1—C2—C7	−179.7 (5)	C15—C10—C11—C12	−0.1 (9)
N1—C1—C6—C5	178.3 (5)	C9—C10—C15—O1	−2.5 (8)
C2—C1—C6—C5	1.2 (8)	C9—C10—C15—C14	178.0 (5)
N1—C1—C2—C3	−177.0 (4)	C11—C10—C15—O1	179.8 (5)
C1—C2—C3—C4	−1.1 (7)	C11—C10—C15—C14	0.3 (8)
C7—C2—C3—C4	178.7 (5)	C10—C11—C12—C13	0.0 (10)
C7—C2—C3—C8	0.2 (8)	C11—C12—C13—C14	−0.1 (11)
C1—C2—C3—C8	−179.5 (5)	C12—C13—C14—C15	0.3 (10)
C2—C3—C4—C5	0.9 (8)	C13—C14—C15—O1	−179.9 (6)
C8—C3—C4—C5	179.3 (5)	C13—C14—C15—C10	−0.4 (9)

Symmetry codes: (i) −*x*+1/2, −*y*+1, *z*+1/2; (ii) *x*+1/2, −*y*+1/2, −*z*; (iii) *x*−1/2, −*y*+1/2, −*z*; (iv) −*x*−1/2, −*y*+1, *z*−1/2; (v) −*x*, *y*−1/2, −*z*−1/2; (vi) −*x*, *y*+1/2, −*z*−1/2; (vii) −*x*+1/2, −*y*+1, *z*−1/2; (viii) −*x*−1/2, −*y*+1, *z*+1/2.

### Hydrogen-bond geometry (Å, °)

**Table:**

Cg1 is the centroid of the C1–C6 ring.

**Table:**

*D*—H···*A*	*D*—H	H···*A*	*D*···*A*	*D*—H···*A*
O1—H1···N1	0.82	1.85	2.582 (6)	148
C7—H7A···Cg1^vii^	0.96	2.92	3.782 (6)	150

Symmetry codes: (vii) −*x*+1/2, −*y*+1, *z*−1/2.

## References

[bb1] Bernstein, J., Davis, R. E., Shimoni, L. & Chang, N.-L. (1995). *Angew. Chem. Int. Ed. Engl.* **34**, 1555–1573.

[bb2] Bruker (2005). *SADABS* Bruker AXS Inc., Madison, Wisconsin, USA.

[bb3] Bruker (2009). *APEX2* and *SAINT* Bruker AXS Inc., Madison, Wisconsin, USA.

[bb4] Farrugia, L. J. (1997). *J. Appl. Cryst.* **30**, 565.

[bb5] Farrugia, L. J. (1999). *J. Appl. Cryst.* **32**, 837–838.

[bb6] Sheldrick, G. M. (2008). *Acta Cryst.* A**64**, 112–122. 10.1107/S0108767307043930 18156677

[bb7] Spek, A. L. (2009). *Acta Cryst.* D**65**, 148–155. 10.1107/S090744490804362X PMC2631630 19171970

[bb8] Tahir, M. N., Tariq, M. I., Ahmad, S., Sarfraz, M. & Ather, A. Q. (2010*a*). *Acta Cryst.* E**66**, o1562. 10.1107/S1600536810020933 PMC3007017 21587805

[bb9] Tahir, M. N., Tariq, M. I., Ahmad, S., Sarfraz, M. & Ather, A. Q. (2010*b*). *Acta Cryst.* E**66**, o1817. 10.1107/S1600536810024165 PMC3006792 21588024

[bb10] Tariq, M. I., Ahmad, S., Tahir, M. N., Sarfaraz, M. & Hussain, I. (2010). *Acta Cryst.* E**66**, o1561. 10.1107/S160053681001932X PMC3006706 21587804

